# Supplementary material for: Min waves without MinC can pattern FtsA-anchored FtsZ filaments on model membranes
Source: Commun Biol. 2022 Jul 7;5:675. doi: 10.1038/s42003-022-03640-1 (PMC9262947; doi:10.1038/s42003-022-03640-1)
Supplement: Supplementary file 2 — Supplementary Information [file 42003_2022_3640_MOESM2_ESM.pdf]

Supplementary Information for

Min waves without MinC can pattern FtsA-anchored FtsZ filaments on model membranes

Elisa Godino, Anne Doerr, Christophe Danelon*

Department of Bionanoscience, Kavli Institute of Nanoscience, Delft University of Technology, Van der Maasweg 9, 2629HZ, Delft, The Netherlands.

*Correspondence: Christophe Danelon

Email: c.j.a.danelon@tudelft.nl

Supplemental information includes:

Supplementary Methods
Figures S1 to S9
Tables S1 to S2

Other supplementary materials for this manuscript:

Movies S1 to S7 are provided as separate files

Supplementary Methods

DNA sequence of the *minD-minE* construct

TAATACGACTCACTATAGGGGAATTGTGAGCGGATAACAATTCCCCTCTAGAAATAATTTTGTTTAACTT
TAAGAAGGAGATATACATATGGCACGCATTATTGTTGTTACTTCGGGCAAAGGGGGTGTTGGTAAGACA
ACCTCCAGCGCGGCCATCGCCACTGGTTTGGCCCAGAAGGGAAAGAAAACTGTCGTGATAGATTTTGA
TATCGGCCTGCGTAATCTCGACCTGATTATGGGTTGTGAACGCCGGGTCGTTTACGATTTCGTCAACGT
CATTCAGGGCGATGCAACGCTAAATCAGGCGTTAATTAAAGATAAGCGTACTGAAAATCTCTATATTCTG
CCGGCATCGCAAACACGCGATAAAGATGCCCTCACCCGTGAAGGGGTCGCCAAAGTTCTTGATGATCT
GAAAGCGATGGATTTTGAATTTATCGTTTGTGACTCCCCGGCAGGGATTGAAACCGGTGCGTTAATGGC
ACTCTATTTTGCAGACGAAGCCATTATTACCACCAACCCGGAAGTCTCCTCAGTACGCGACTCTGACCG
TATTTTAGGCATTCTGGCGTCGAAATCACGCCGCGCAGAAAATGGCGAAGAGCCTATTAAAGAGCACCT
GCTGTTAACGCGCTATAACCCAGGCCGCGTAAGCAGAGGTGACATGCTGAGCATGGAAGATGTGCTG
GAGATCCTGCGCATCAAACTCGTCGGCGTGATCCCAGAGGATCAATCAGTATTGCGCGCCTCTAACCA
GGGTGAACCGGTCATTCTCGACATTAACGCCGATGCGGGTAAAGCCTACGCAGATACCGTAGAACGTC
TGTTGGGAGAAGAACGTCCTTTCCGCTTCATTGAAGAAGAGAAGAAAGGCTTCCTCAAACGCTTGTTCG
GAGGATAAGGATCCGGCTGCTAACAAAGCCCGAAAGGAAGCTGAGTTGGCTGCTGCCACCGCTGAGC
AATAACTAGCATAACCCCTTGGGGCCTCTAAACGGGTCTTGAGGGGTTTTTTGATTGGGTATCGGATCC
CGGGCCCGTCGACTGCAGAGGCCTGCATGCAAGCTTGGCGTAATCATGGTCATAGCTGTTTCCTGTGT
GTAATACGACTCACTATAGGGGAATTGTGAGCGGATAACAATTCCCCTCTAGAAATAATTTTGTTTAACT
TTAAGAAGGAGATATACATATGGCGCTGCTGGATTTCTTTCTGAGCCGTAAGAAAAACACCGCGAACAT
CGCGAAAGAGCGTCTGCAAATCATTGTTGCGGAGCGTCGTCGTAGCGATGCGGAACCGCACTACCTG
CCGCAGCTGCGTAAAGATATCCTGGAAGTGATTTGCAAGTATGTTCAAATTGACCCGGAGATGGTGACC
GTTCAGCTGGAACAAAAGGACGGTGATATCAGCATTCTGGAGCTGAACGTTACCCTGCCGGAAGCGGA
GGAACTGAAGTAAGGATCCGGCTGCTAACAAAGCCCGAAAGGAAGCTGAGTTGGCTGCTGCCACCGC
TGAGCAATAACTAGCATAACCCCTTGGGGCCTCTAAACGGGTCTTGAGGGGTTTTTTG

DNA sequence of the *ftsA-minD-minE* construct

TAATACGACTCACTATAGGGGAATTGTGAGCGGATAACAATTCCCCTCTAGAAATAATTTTGTTTAACTT
TAAGAAGGAGATATACATATGATCAAGGCGACCGACCGTAAGCTGGTTGTGGGCCTGGAGATTGGCAC
CGCGAAGGTTGCGGCGCTGGTTGGCGAGGTTCTGCCGGATGGTATGGTTAACATTATCGGCGTTGGTA
GCTGCCCGAGCCGTGGCATGGACAAAGGTGGTGTGAACGACCTGGAAAGCGTGGTTAAGTGCGTGCA
GCGTGCGATTGACCAGGCGGAGCTGATGGCGGACTGCCAAATCAGCAGCGTTTACCTGGCGCTGAGC
GGCAAGCACATCAGCTGCCAAAACGAGATTGGTATGGTGCCGATTAGCGAAGAGGAAGTTACCCAGGA
AGATGTGGAGAACGTGGTTCACACCGCGAAAAGCGTTCGTGTGCGTGATGAACACCGTGTGCTGCACG
TTATCCCGCAAGAATACGCGATCGATTACCAGGAAGGTATCAAAAACCCGGTTGGTCTGAGCGGTGTT
CGTATGCAGGCGAAAGTGCACCTGATTACCTGCCACAACGATATGGCGAAGAACATTGTGAAAGCGGT
TGAACGTTGCGGTCTGAAGGTTGACCAGCTGATCTTCGCGGGTCTGGCGAGCAGCTACAGCGTTCTGA
CCGAAGATGAGCGTGAACTGGGTGTTTGCGTTGTGGATATCGGCGGTGGCACGATGGATATCGCGGT
GTATACCGGTGGCGCGCTGCGTCACACCAAAGTGATTCCGTATGCGGGTAACGTGGTTACCAGCGACA
TCGCGTACGCGTTTGGCACCCCGCCGAGCGATGCGGAGGCGATCAAAGTGCGTCACGGTTGCGCGCT
GGGTAGCATTGTGGGTAAAGATGAGAGCGTGGAAGTTCCGAGCGTTGGTGGCCGTCCGCCGCGTAGC

CTGCAACGTCAGACCCTGGCGGAAGTTATCGAGCCGCGTTACACCGAACTGCTGAACCTGGTGAACGA
AGAGATCCTGCAACTGCAAGAGAAACTGCGTCAGCAAGGTGTTAAGCACCACCTGGCGGCGGGCATT
GTTCTGACCGGCGGTGCGGCGCAGATCGAAGGTCTGGCGGCGTGCGCGCAACGTGTTTTCCACACCC
AAGTTCGTATCGGTGCGCCGCTGAACATCACCGGTCTGACCGATTACGCGCAAGAGCCGTACTATAGC
ACCGCGGTTGGTCTGCTGCACTATGGCAAAGAGAGCCACCTGAACGGCGAGGCGGAAGTGGAGAAGC
GTGTGACCGCGAGCGTTGGTAGCTGGATTAAGCGTCTGAATAGCTGGCTGCGTAAGGAGTTCTAAGGA
TCCGGCTGCTAACAAAGCCCGAAAGGAAGCTGAGTTGGCTGCTGCCACCGCTGAGCAATAACTAGCAT
AACCCCTTGGGGCCTCTAAACGGGTCTTGAGGGGTTTTTTGATCGGATCCCGGGCCCGTCGACTGCAG
AGGCCTGCATGCAAGCTTGGCGTAATCATGGTCATAGCTGTTTCCTGTGTGCTGCCCGCTTTCCAGTCG
GGAAACCTGTCGTGCCAGCTGCATTAATGAATCGGCCAACGCCAGTCACGACGTTGTAAAACGACGGC
CAGTGAATTCGAGCTCGGTACCTCGCGAATGCATCTAGATGCGAAATTAATACGACTCACTATAGGGGA
ATTGTGAGCGGATAACAATTCCCCTCTAGAAATAATTTTGTTTAACTTTAAGAAGGAGATATACATATGG
CACGCATTATTGTTGTTACTTCGGGCAAAGGGGGTGTTGGTAAGACAACCTCCAGCGCGGCCATCGCC
ACTGGTTTGGCCCAGAAGGGAAAGAAAACTGTCGTGATAGATTTTGATATCGGCCTGCGTAATCTCGAC
CTGATTATGGGTTGTGAACGCCGGGTCGTTTACGATTTCGTCAACGTCATTCAGGGCGATGCAACGCTA
AATCAGGCGTTAATTAAAGATAAGCGTACTGAAAATCTCTATATTCTGCCGGCATCGCAAACACGCGAT
AAAGATGCCCTCACCCGTGAAGGGGTCGCCAAAGTTCTTGATGATCTGAAAGCGATGGATTTTGAATTT
ATCGTTTGTGACTCCCCGGCAGGGATTGAAACCGGTGCGTTAATGGCACTCTATTTTGCAGACGAAGC
CATTATTACCACCAACCCGGAAGTCTCCTCAGTACGCGACTCTGACCGTATTTTAGGCATTCTGGCGTC
GAAATCACGCCGCGCAGAAAATGGCGAAGAGCCTATTAAAGAGCACCTGCTGTTAACGCGCTATAACC
CAGGCCGCGTAAGCAGAGGTGACATGCTGAGCATGGAAGATGTGCTGGAGATCCTGCGCATCAAACT
CGTCGGCGTGATCCCAGAGGATCAATCAGTATTGCGCGCCTCTAACCAGGGTGAACCGGTCATTCTCG
ACATTAACGCCGATGCGGGTAAAGCCTACGCAGATACCGTAGAACGTCTGTTGGGAGAAGAACGTCCT
TTCCGCTTCATTGAAGAAGAGAAGAAAGGCTTCCTCAAACGCTTGTTCGGAGGATAAGGATCCGGCTG
CTAACAAAGCCCGAAAGGAAGCTGAGTTGGCTGCTGCCACCGCTGAGCAATAACTAGCATAACCCCTT
GGGGCCTCTAAACGGGTCTTGAGGGGTTTTTTGATTGGGTATCGGATCCCGGGCCCGTCGACTGCAGA
GGCCTGCATGCAAGCTTGGCGTAATCATGGTCATAGCTGTTTCCTGTGTGTAATACGACTCACTATAGG
GGAATTGTGAGCGGATAACAATTCCCCTCTAGAAATAATTTTGTTTAACTTTAAGAAGGAGATATACATA
TGGCGCTGCTGGATTTCTTTCTGAGCCGTAAGAAAAACACCGCGAACATCGCGAAAGAGCGTCTGCAA
ATCATTGTTGCGGAGCGTCGTCGTAGCGATGCGGAACCGCACTACCTGCCGCAGCTGCGTAAAGATAT
CCTGGAAGTGATTTGCAAGTATGTTCAAATTGACCCGGAGATGGTGACCGTTCAGCTGGAACAAAAGG
ACGGTGATATCAGCATTCTGGAGCTGAACGTTACCCTGCCGGAAGCGGAGGAACTGAAGTAAGGATCC
GGCTGCTAACAAAGCCCGAAAGGAAGCTGAGTTGGCTGCTGCCACCGCTGAGCAATAACTAGCATAAC
CCCTTGGGGCCTCTAAACGGGTCTTGAGGGGTTTTTTG

DNA sequence QconCAT

TAATACGACTCACTATAGGGAGACCACAACGGTTTCCCTCTAGAAATAATTTTGTTTAACTTTAAGAAGG
AGATATACATATGCGGGGTTCTCATCATCATCATCATCATGGTATGGCTAGCATGACTGGTGGACAGCA
AATGGGTCGGGACCTGTACGACGACGACGATAAGAGCCTGTTTCCGAAAAGCAGCGTGGCGGTTAGC
TGGATTGGTAAAGCGGCGGCGGCGGGTGTGAGCGATGATGCGCGTATGGACCTGGCGAGCCAGTTTT
ACGAGAAGGCGGTGCTGGTTGATCTGGAACCGGGTGTTATTGCGCGTGCGACCAACCCGCTGGTGCG

TGCGGCGAGCCTGCTGGTTGACAAACTGGGCGAGAGCAGCAGCGGTAGCTATGTTCCGCGTAACATC
ATGGGTATTGAGGTGCCGGAAACCCTGGTTCACAAGACCATCTTCTTTAAACAGGCGAGCGAACAAAA
CTGGGCGAACTACAGCGCGGAGCAGAACCGTTTCGAAGGCGATACCCTGGTGAACCGTACCTACATCA
TCAGCATCCTGTTCAAGAACCAGATTGCGACCCTGGCGCAAAGCTATCGTGGTCTGGGTGCGGGTGCG
AACCCGGAAGTGGGTCGTGAATATCAAGACATTATCCGTATGCTGCAGCAAACCATCGAGCAGGCGCT
GCTGGAACAAGGTCGTCTGATTGTGCTGCTGCTGCTGGGCTTCGGCGAGCGTGCGTACGCGGATACC
GTTGAACGTGGCAGCAGCTTTACCCTGAGCGTGGTTCACCTGCACGAGGCGGAACCGAAGCTGCAGT
GGCAAGAGAGCGACGGTACCATCAAATACCGTCCGACCGACGATTTCGATGCGCGTTATACCGAACTG
CTGAACCTGGTGAACGAGGAAATTCTGCAGCTGCAAGAGAAGGACATCCTGGAAGTTATTTGCAAAGAT
GCGCTGAACCAGGCGGCGGACGATCTGAACCAACGTGTGGTTAACGACAACGCGCCGCAGACCGCGA
AAGATGCGCTGAGCCTGGCGCGTGGCGGTGTGAACGACCTGGAGAGCGTGGTTAAGATTATCGTGGT
TACCAGCGGTAAAGCGCTGGCGGGTGCGAGCGGTGACCGTGATACCATCGGCGACATTATCATTCTG
CCGCGTCTGAGCGATTACGGTGTTCAACTGCGTGCGCCGGTGGTTGTTCCGGCGGGTGTGGACGTGA
AAGCGATTAGCCTGAGCGTGCGTTAACTAGCATAACCCCTTGGGGCCTCTAAACGGGTCTTGAGGGGT
TTTTTG

DNA sequence of the *ftsA construct**

TAATACGACTCACTATAGGGGAATTGTGAGCGGATAACAATTCCCCTCTAGAAATAATTTTGTTTAACTT
TAAGAAGGAGATATACATATGATCAAGGCGACCGACCGTAAGCTGGTTGTGGGCCTGGAGATTGGCAC
CGCGAAGGTTGCGGCGCTGGTTGGCGAGGTTCTGCCGGATGGTATGGTTAACATTATCGGCGTTGGTA
GCTGCCCGAGCCGTGGCATGGACAAAGGTGGTGTGAACGACCTGGAAAGCGTGGTTAAGTGCGTGCA
GCGTGCGATTGACCAGGCGGAGCTGATGGCGGACTGCCAAATCAGCAGCGTTTACCTGGCGCTGAGC
GGCAAGCACATCAGCTGCCAAAACGAGATTGGTATGGTGCCGATTAGCGAAGAGGAAGTTACCCAGGA
AGATGTGGAGAACGTGGTTCACACCGCGAAAAGCGTTCGTGTGCGTGATGAACACCGTGTGCTGCACG
TTATCCCGCAAGAATACGCGATCGATTACCAGGAAGGTATCAAAAACCCGGTTGGTCTGAGCGGTGTT
CGTATGCAGGCGAAAGTGCACCTGATTACCTGCCACAACGATATGGCGAAGAACATTGTGAAAGCGGT
TGAACGTTGCGGTCTGAAGGTTGACCAGCTGATCTTCGCGGGTCTGGCGAGCAGCTACAGCGTTCTGA
CCGAAGATGAGCGTGAACTGGGTGTTTGCGTTGTGGATATCGGCGGTGGCACGATGGATATCGCGGT
GTATACCGGTGGCGCGCTGCGTCACACCAAAGTGATTCCGTATGCGGGTAACGTGGTTACCAGCGACA
TCGCGTACGCGTTTGGCACCCCGCCGAGCGATGCGGAGGCGATCAAAGTGCGTCACGGTTGCGCGCT
GGGTAGCATTGTGGGTAAAGATGAGAGCGTGGAAGTTCCGAGCGTTGGTGGCCGTCCGCCGCGTAGC
CTGCAACGTCAGACCCTGGCGGAAGTTATCGAGCCGCGTTACACCGAACTGCTGAACCTGGTGAACGA
AGAGATCCTGCAACTGCAAGAGAAACTGCGTCAGCAAGGTGTTAAGCACCACCTGGCGGCGGGCATT
GTTCTGACCGGCGGTGCGGCGCAGATCGAAGGTCTGGCGGCGTGCGCGCAACGTGTTTTCCACACCC
AAGTTCGTATCGGTGCGCCGCTGAACATCACCGGTCTGACCGATTACGCGCAAGAGCCGTACTATAGC
ACCGCGGTTGGTCTGCTGCACTATGGCAAAGAGAGCCACCTGAACGGCGAGGCGGAAGTGGAGAAGC
GTGTGACCGCGAGCGTTGGTAGCTGGATTAAGCGTCTGAATAGCTGGCTGCGTAAGGAGTTCTAAGGA
TCCGGCTGCTAACAAAGCCCGAAAGGAAGCTGAGTTGGCTGCTGCCACCGCTGAGCAATAACTAGCAT
AACCCCTTGGGGCCTCTAAACGGGTCTTGAGGGGTTTTTTG

DNA sequence of the *zipA* construct

TAATACGACTCACTATAGGGGAATTGTGAGCGGATAACAATTCCCCTCTAGAAATAATTTTGTTTAACTT
TAAGAAGGAGATATACATATGCATCACCATCATCACCATCACGGTTTTTGGACCAGCCGCAAAGAACGT
AGCAGCATGTTCCGTGATCGTCCGCTGAAGCGTATGAAGAGCAAACGTGACGATGACAGCTACGATGA
GGACGTGGAAGATGACGAGGGTGTTGGCGAGGTGCGCGTTCATCGTGTGAACCATGCGCCGGCGAAC
GCGCAGGAGCACGAAGCGGCGCGTCCGAGCCCGCAGCACCAATATCAGCCGCCGTATGCGAGCGCG
CAACCGCGTCAGCCGGTGCAGCAACCGCCGGAAGCGCAAGTTCCGCCGCAGCATGCGCCGCACCCG
GCGCAACCGGTTCAGCAACCGGCGTATCAACCGCAACCGGAGCAGCCGCTGCAACAACCGGTGAGCC
CGCAAGTTGCGCCGGCGCCGCAGCCGGTGCACAGCGCGCCGCAACCGGCGCAGCAAGCGTTCCAAC
CGGCGGAACCGGTTGCGGCGCCGCAGCCGGAACCGGTTGCGGAGCCGGCGCCGGTTATGGACAAGC
CGAAACGTAAAGAGGCGGTGATCATTATGAACGTTGCGGCGCACCACGGTAGCGAACTGAACGGCGA
GCTGCTGCTGAACAGCATCCAGCAAGCGGGTTTCATTTTTGGCGATATGAACATCTACCACCGTCATCT
GAGCCCGGATGGTAGCGGTCCGGCGCTGTTTAGCCTGGCGAACATGGTGAAACCGGGCACCTTCGAT
CCGGAAATGAAGGACTTTACCACCCCGGGTGTGACCATTTTCATGCAAGTTCCGAGCTATGGCGATGA
GCTGCAAAACTTTAAACTGATGCTGCAAAGCGCGCAGCACATCGCGGACGAGGTTGGTGGCGTGGTTC
TGGATGACCAGCGTCGTATGATGACCCCGCAAAAGCTGCGTGAATACCAGGACATTATCCGTGAAGTG
AAAGATGCGAACGCGTAAGGATCCGGCTGCTAACAAAGCCCGAAAGGAAGCTGAGTTGGCTGCTGCC
ACCGCTGAGCAATAACTAGCATAACCCCTTGGGGCCTCTAAACGGGTCTTGAGGGGTTTTTTG

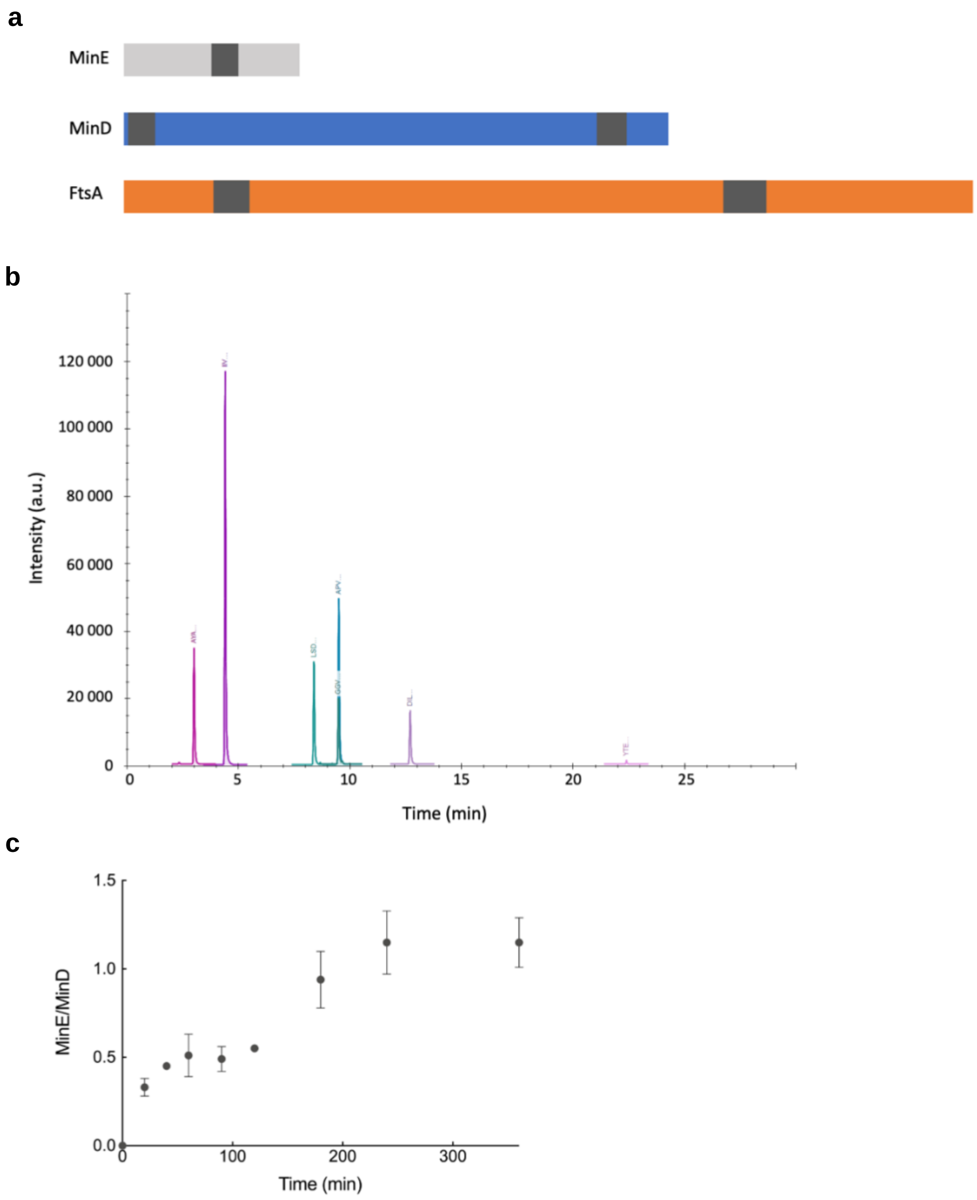

Figure S1. Quantitative LC-MS analysis of cell-free synthesized FtsA, MinD and MinE in bulk reactions. **a** Cartoon depicting the position of the analyzed specific proteolytic peptides (dark grey domains) along the primary sequence of FtsA, MinD and MinE, from N-term (left) to C-term (right). **b** LC-MS chromatogram of the investigated peptides. From left to right: AYADTVER (MinD); IIVVTSGK (MinD); LSDYGVQLR (ribosomal protein S4); GGVNDLESVVK (FtsA); APVVVPAGVDVK (ribosomal protein L6); DILEVICK (MinE); YTELLNLVNEEILQLQEK (FtsA). The two peptides for ribosomal proteins were used for quality control. **c** Concentration ratio of expressed MinE and MinD proteins as a function of time. Symbols correspond to mean values and the error bars represent standard deviations.

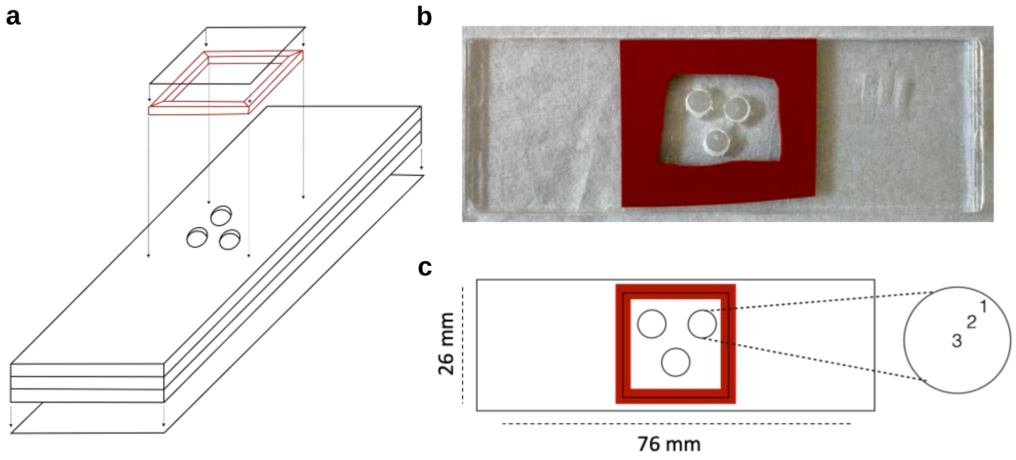

Figure S2. Home-made imaging glass chamber. **a** Schematic of the chamber assembly. Each well has a diameter of 2.5 mm. Dashed arrows indicate the direction of assembly of each part of the chamber. Three glued and drilled glass slides were attached to a 150-µm thick glass coverslip using NOA 61 glue to form the bottom of the chamber. A 20×20-mm glass coverslip was placed on top of a double-sided adhesive silicone sheet (in red) to close the chamber. **b** Picture of an imaging chamber taken from the top. **c** Schematic of the sealed chamber viewed from the top. The digits (1, 2, 3) indicate the position of the fields of view as displayed in Figure 2b and 3b: (3) indicates the center of the chamber, (1) the edges of the chamber and (2) the intermediate areas.

a

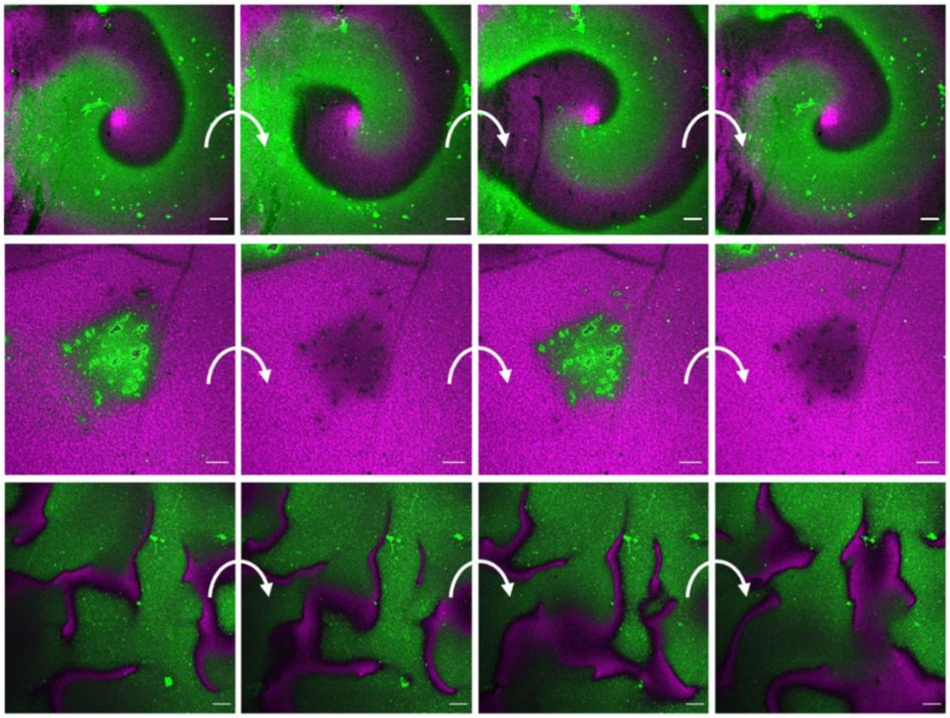

b

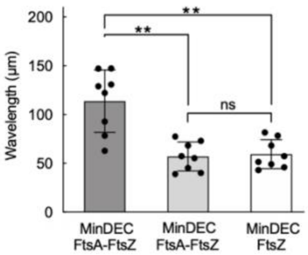

Figure S3. MinDEC patterns and FtsA-FtsZ structures in supported membrane assays. **a** Composite fluorescence microscopy images of FtsA-FtsZ and MinDEC dynamic patterns on an SLB. Different patterns including spirals (top), standing (middle) and random (bottom) are shown. Arrows indicate time lapse between images. Experimental conditions and color coding are the same as in main text Figure 2b and 2c. Scale bars are 10 µm. **b** Calculated wavelengths for MinDEC waves in areas where FtsA-FtsZ co-existed (dark grey), were excluded (light grey), and without expressed FtsA (white). Data are from three biological repeats and two to three fields of view have been analyzed per sample. Bar height represents the mean value and the error bar corresponds to the standard deviation. Symbols are values for individual fields of view aggregated from three biological replicates. Values obtained for different conditions were statistically compared by performing a two-tailed Welch's *t*-test. Asterisks indicate *P* value <0.01, while 'ns' denotes a non-significant difference with *P* value >0.05.

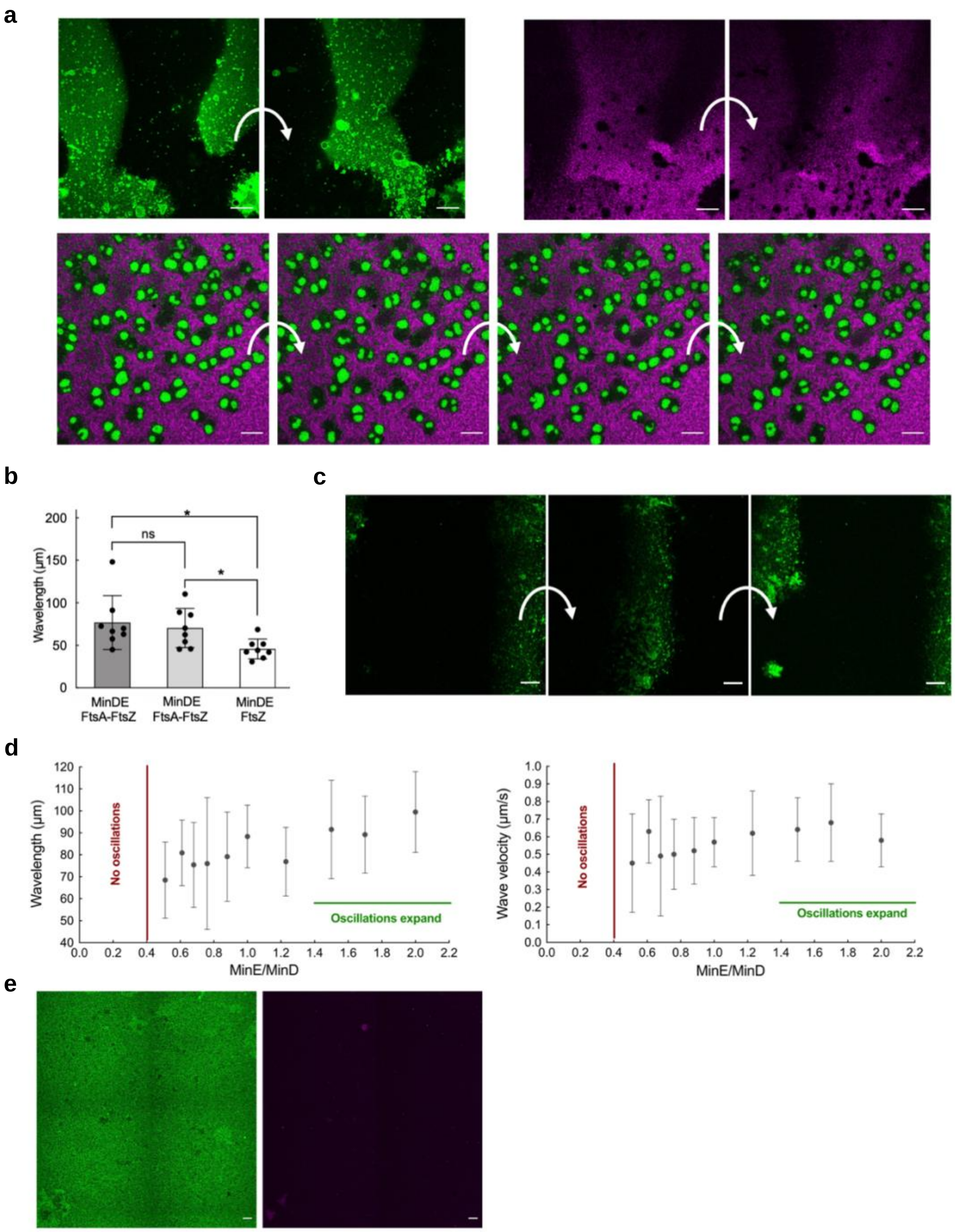

Figure S4. Self-organized MinDE proteins, without MinC, can effectively rearrange FtsA-anchored FtsZ filaments on supported membranes. **a** Fluorescence microscopy images showing that MinDE dynamic patterns rearrange the FtsA-FtsZ cytoskeletal structures on an SLB. Examples of planar (top, split channels) and standing (bottom, merged channels) waves are shown. Arrows indicate time lapse between images. Experimental conditions and color coding are the same as in main text Figure 3, with eGFP-MinD in green and FtsZ-A647 in magenta. Scale bars are 10 μm. **b** Calculated wavelengths for MinDE waves in areas where FtsA-FtsZ co-existed (dark grey), were excluded

(light grey), and without expressed FtsA (white). Data are from three biological repeats and two to three fields of view have been analyzed per sample. Bar height represents the mean value and the error bar corresponds to the standard deviation. Symbols are values for individual fields of view aggregated from three biological replicates. Values obtained for different conditions were statistically compared by performing a two-tailed Welch's *t*-test. Asterisks indicate *P* value <0.05, while 'ns' denotes a non-significant difference. **c** Fluorescence microscopy images of FtsA (0.4 µM FtsA-A488, green signal) dynamic patterns driven by MinDE on an SLB. Sharp propagating waves of FtsA are visible. Arrows indicate time lapse between images. FtsA-A488 (green), scale bars are 10 µm. **d** Wavelength (left) and velocity (right) of MinDE oscillations for different MinE-to-MinD protein ratios. At MinE/D ratios <0.44, no oscillations were observed. At MinE/D ratios >1.4, the oscillations that were mostly confined to the edges of the chamber (see Figure 3b) expanded over larger membrane areas. **e** Fluorescence microscopy images showing that MinD and FtsZ do not directly interact on the SLB. Here, only MinD was expressed (no FtsA and no MinE). Color coding: eGFP-MinD (green), FtsZ-A647 (magenta). Scale bars are 10 µm.

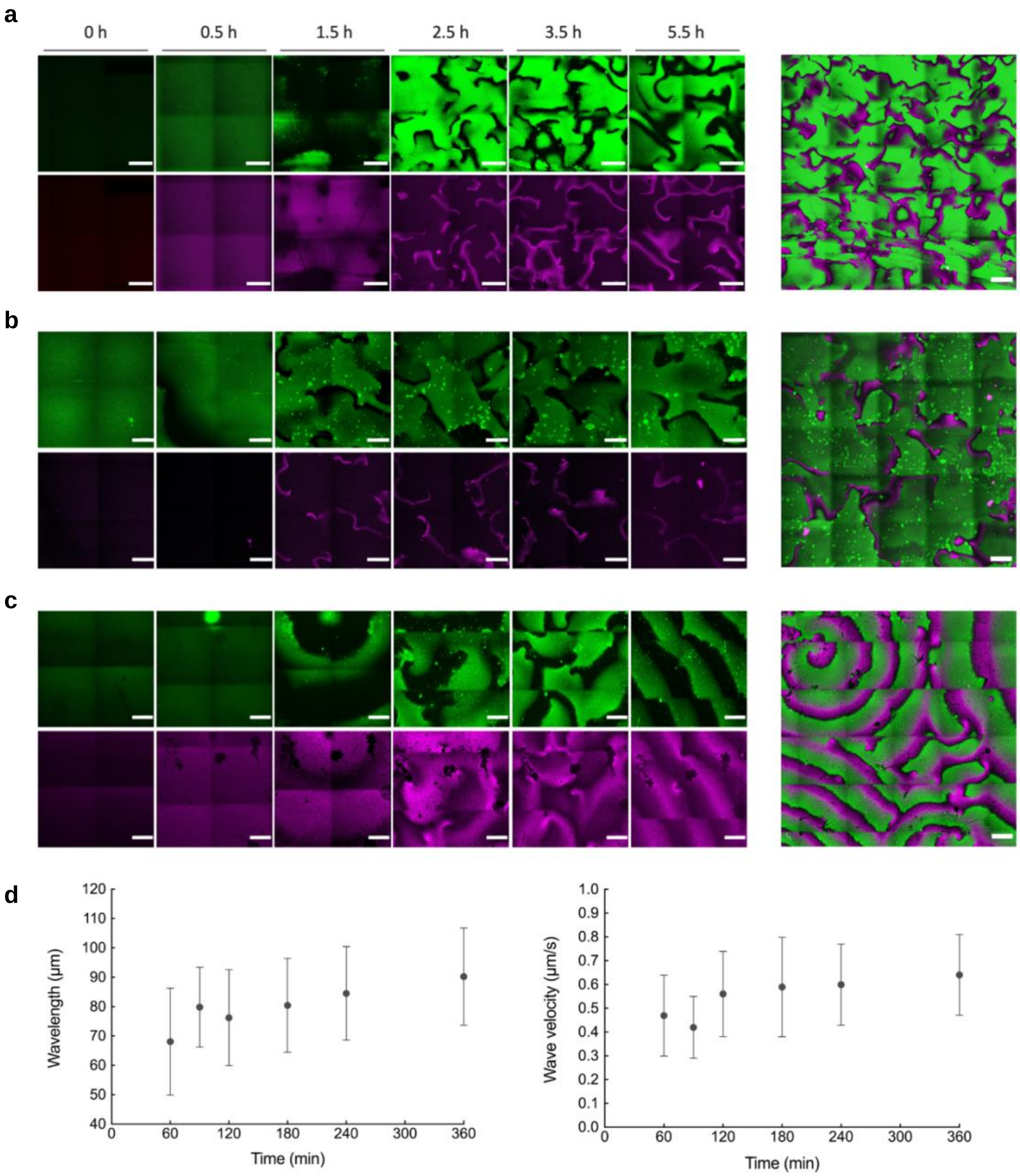

Figure S5. Dynamic patterning of MinDE(C) and FtsA-FtsZ during in situ co-expression of FtsA, MinD and MinE. Samples were monitored up to 5.5 hours to study pattern creation and temporal evolution. **a** Two-by-two tile scan microscope images showing medium-scale organization of FtsA-FtsZ and MinDEC dynamic patterns at different time points. A mosaic image of 5×5 fields of view acquired after 3.5 hours of expression reveals large-scale, uniformly distributed waves of FtsA-FtsZ and MinDEC. Signals from eGFP-MinC and FtsZ-A647 are in green and magenta, respectively. The two channels were overlaid to compose the large mosaic image. Scale bars are 20 µm and 50 µm for the 2×2 and 5×5 tile scans, respectively. **b** Same as in **a** except that eGFP-MinC was replaced by a trace amount of purified eGFP-MinD (green). **c** Same as in **b** but in this sample the protein network self-organized into spirals and planar waves.

Dynamic FtsA-FtsZ patterns anti-correlating with the traveling MinDE(C) waves formed uniformly across the chamber. The coexistence of the two subsystems over the entire membrane surface contrasts with the spatial segregation of Min waves and FtsA-anchored FtsZ filaments when pre-expressed proteins were added to the imaging chamber (main text Figure 2b and Figure 3b). This different behavior may be attributed to protein-specific expression kinetics, which influences the coupling of the two subsystems at large length scales. For instance, MinE production being slow (main text Figure 1c) enables MinD and FtsA to bind homogenously all over the available membrane within the first hour until MinE concentration rises, triggering wave formation. Furthermore, in situ protein biogenesis may occur in close proximity to the lipid bilayer due to the intrinsic ability of ribosomes to bind to membranes, which potentially results in a different local concentration of FtsA and MinD comparing with pre-expressed proteins. In the presence of eGFP-MinC, rings of FtsA-FtsZ cytoskeletal structures formed after 1 hour of gene expression, concomitant to the emergence of MinDEC patterns that began in sporadic areas as standing waves (**a**). In the course of protein production, MinDEC self-organized into sharper traveling waves that colonized the whole chamber, causing stronger patterning of membrane-recruited FtsZ (**a**). In the assays conducted without MinC, eGFP-MinD was recruited to the membrane at the start of the reaction (**b** and **c**). After ~30 min, extensive dynamic patterns of MinDE appeared, followed by FtsZ surface waves that became more pronounced as more FtsA was synthesized. After 1 to 1.5 hours of gene expression, the anticorrelated concentration gradients of membrane-bound FtsA-FtsZ and MinDE became well defined. Here too, MinDE self-organized into crisper traveling waves as protein synthesis was progressing. Together, these results demonstrate that in situ cell-free gene expression can be exploited for timing protein-protein and protein-membrane interactions, which modulates the large-scale organization of the coupled Min-FtsA-FtsZ system. **d** Wavelength (left) and velocity (right) of Min waves during in situ co-expression of FtsA, MinD and MinE. Oscillation properties do not notably change over time.

a

b

c

d

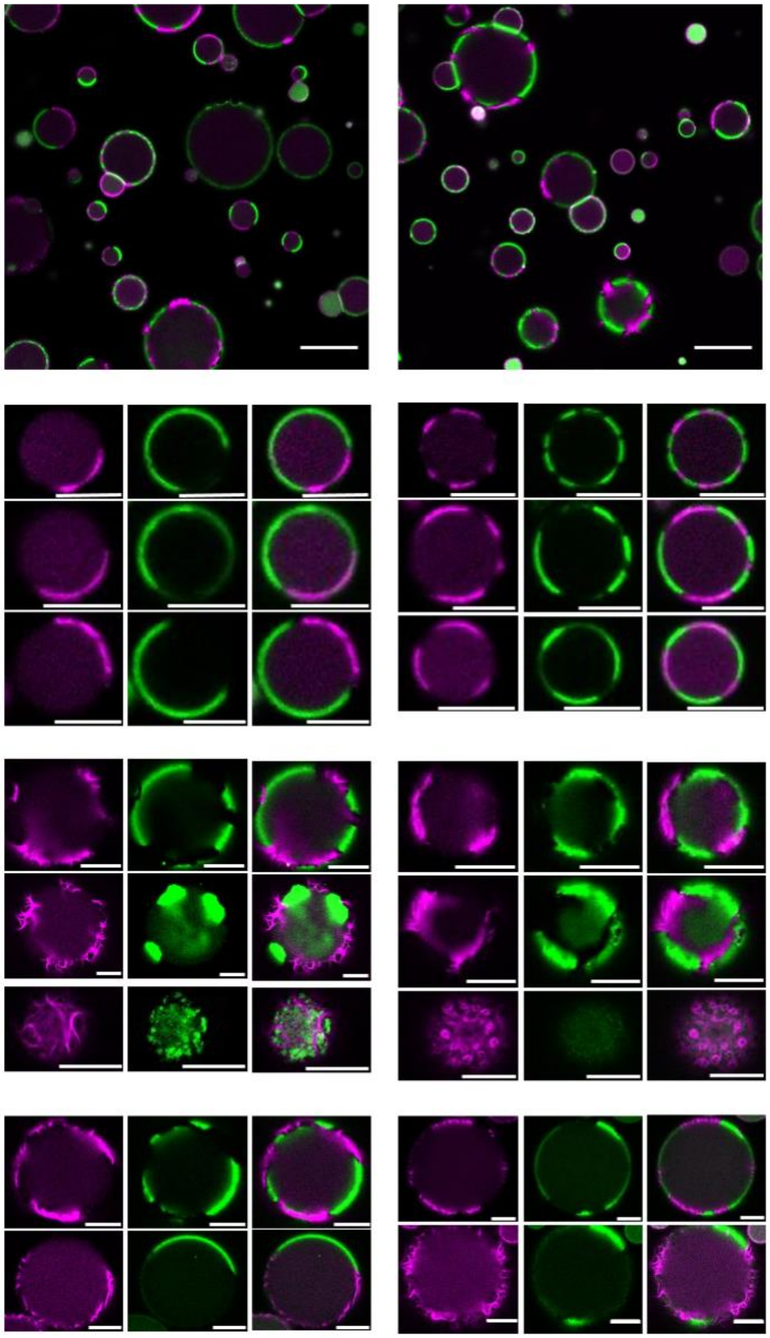

Figure S6. Reconstitution of MinDEC and FtsA-FtsZ subsystems in water-in-oil droplets. Composite fluorescence microscopy images of droplet populations and a library of single droplet images with reconstituted MinDEC patterns and FtsA-FtsZ subsystems are shown. **a** Composite image of droplet population. Experimental conditions were as described in main text Figure 4b. **b** Fluorescence images of droplets exhibiting antiphase dynamic patterns of MinDEC and FtsA-FtsZ. Experimental conditions were as described in main text Figure 4b. **c** Same as in **a**, but images were acquired closer to the dome of the droplets. Multiple interfacial FtsZ polarization sites rearranged by the Min dynamics are visible. Experimental conditions were as described in main text Figure 4b. **d** Same as in **b-c**, except that bigger droplets (diameter >20 µm) were imaged. Here, FtsZ cytoskeletal structures are clearly resolved. **a-d** Experimental conditions were as described in main text Figure 4b. Color coding: eGFP-MinC (green), FtsZ-A647 (magenta). Scale bars are 10 µm.

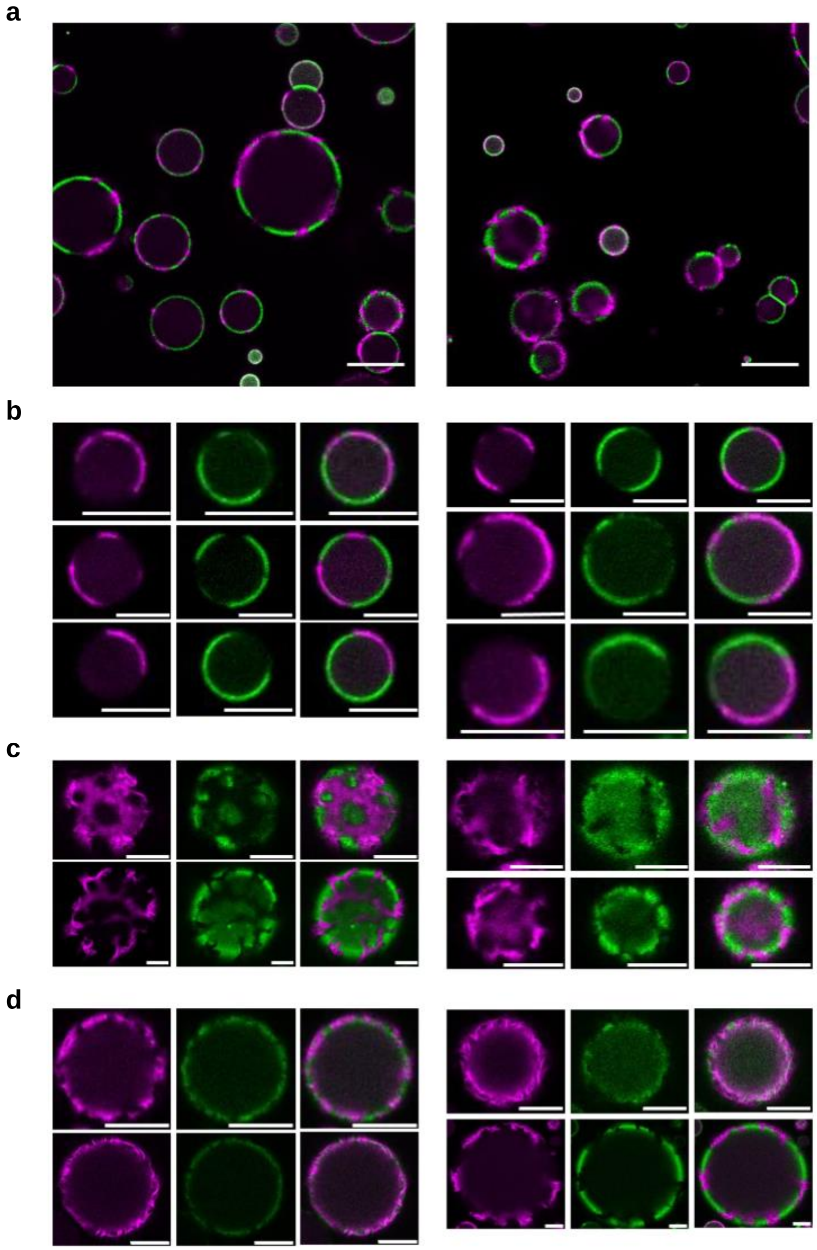

Figure S7. Reconstitution of MinDE and FtsA-FtsZ subsystems in water-in-oil droplets. Composite fluorescence microscopy images of droplet populations and a library of single droplet images with reconstituted MinDE patterns and FtsA-FtsZ subsystems are shown. **a** Composite image of droplet population. Experimental conditions were as described in main text Figure 4c. **b** Fluorescence images of droplets exhibiting antiphase dynamic patterns of MinDE and FtsA-FtsZ. Experimental conditions were as described in main text Figure 4c. **c** Same as in **a**, but images were acquired closer to the dome of the droplets. Multiple interfacial FtsZ polarization sites rearranged by the Min dynamics are visible. Experimental conditions were as described in main text Figure 4c. **d** Same as in **b-c**, except that bigger droplets (diameter >20 µm) were imaged. Here, FtsZ cytoskeletal structures are clearly resolved. Experimental conditions were as described in main text Figure 4c. **a-d** Color coding: eGFP-MinD (green), FtsZ-A647 (magenta). Scale bars are 10 µm.

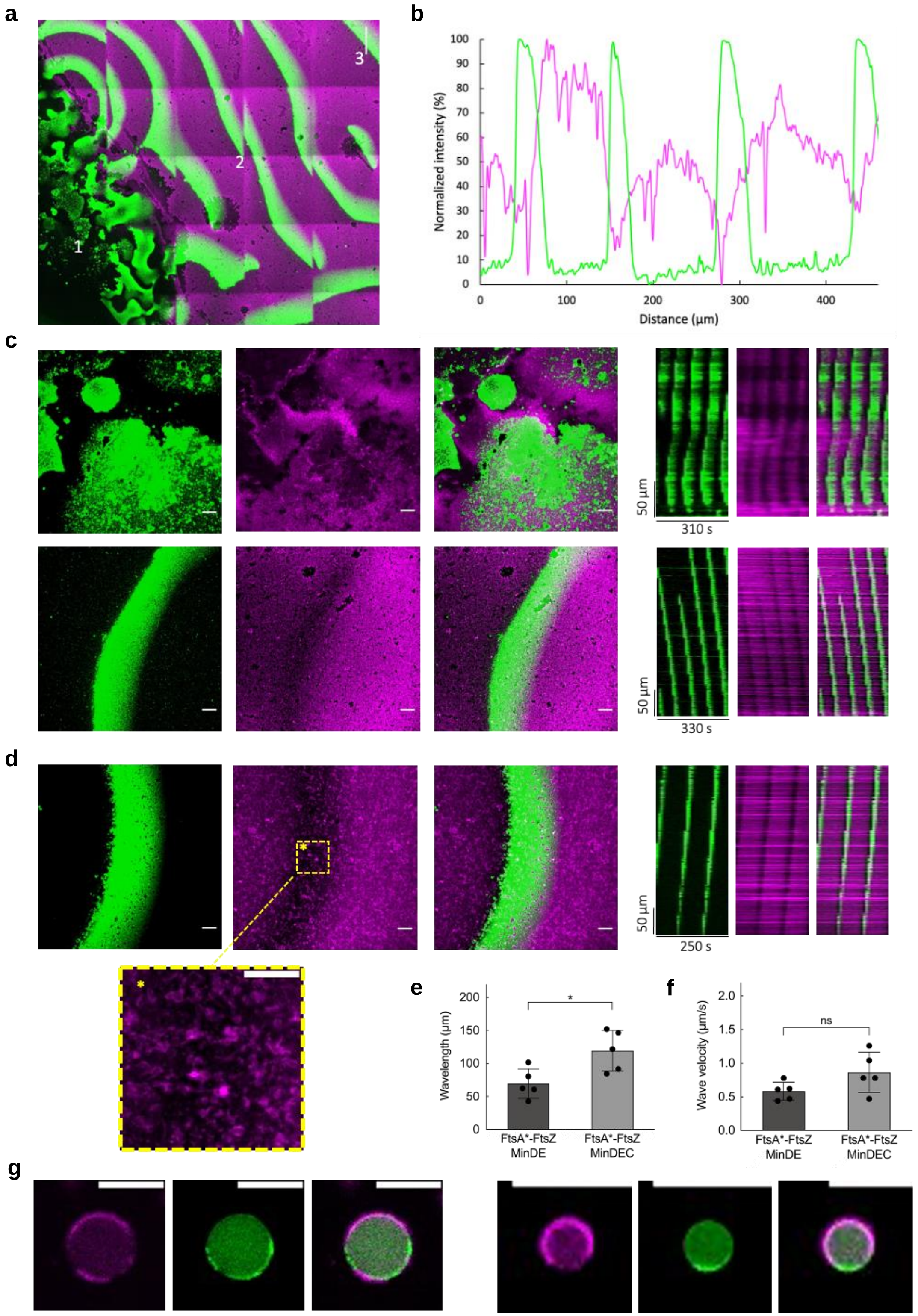

Figure S8. MinDE(C) proteins regulate FtsA*-FtsZ patterns on supported membranes. a Mosaic of 5×5 tile scan microscope images showing large-scale organization of FtsA*-FtsZ and MinDE dynamic patterns. MinDE oscillating gradients dominate at the edges of the chamber (1), while FtsA*-anchored FtsZ filaments are confined at the center of the chamber (3). Between these

two areas (2), dynamic patterns of the two subsystems can be seen. Interestingly, the area where the two subsystems co-exist is extended towards the center of the chamber (3) when FtsA is substituted with FtsA*. Signals from eGFP-MinD and FtsZ-A647 are in green and magenta, respectively. Composite images of overlaid channels are shown. Scale bar is 50 μm. **b** Intensity profiles of MinD and FtsZ are shown. Color coding is the same as in the microscopy image. **c** Fluorescence microscopy images of FtsA*-FtsZ and MinDE dynamic patterns acquired in the intermediate SLB area. Different oscillation patterns were observed: sharp propagating waves of FtsA*-FtsZ and MinDE (top), and low-amplitude oscillations of FtsZ anticorrelating with MinDE patterns (bottom). Signals from eGFP-MinD and FtsZ-A647 are in green and magenta, respectively Scale bars are 10 μm. For both fields of view, the time evolution of the oscillations was analyzed, and kymographs were constructed (as displayed on the side). Color coding is the same as in the microscopy images. **d** Fluorescence microscopy images of FtsA*-FtsZ and MinDEC dynamic patterns. Even in the presence of MinC, the displacement of FtsA*-anchored FtsZ from the membrane was not complete. A zoom-in image of the framed area in the FtsZ channel is also displayed, revealing that some curved FtsZ filaments remain tethered as the Min wave propagates. Signals from eGFP-MinC and FtsZ-A647 are in green and magenta, respectively. Scale bars are 10 μm. The corresponding kymographs are displayed. Color coding is the same as in the microscopy images. **e-f** Calculated wavelength (**e**) and velocity (**f**) of MinDE and MinDEC waves, both in the presence of FtsA*-FtsZ. Data are from two biological replicates and two to three fields of view were analyzed per sample. Bar height represents the mean value and the standard deviation is appended. Symbols are values for individual fields of view aggregated from the biological replicates. Values obtained for different conditions were statistically compared by performing a two-tailed Welch's *t*-test. Asterisk indicates *P* value <0.05, while 'ns' denotes a non-significant difference. **g** Fluorescence images of droplets exhibiting antiphase dynamic patterns of MinDE and FtsA*-FtsZ. Color coding: eGFP-MinD (green), FtsZ-A647 (magenta). Scale bars are 10 μm.

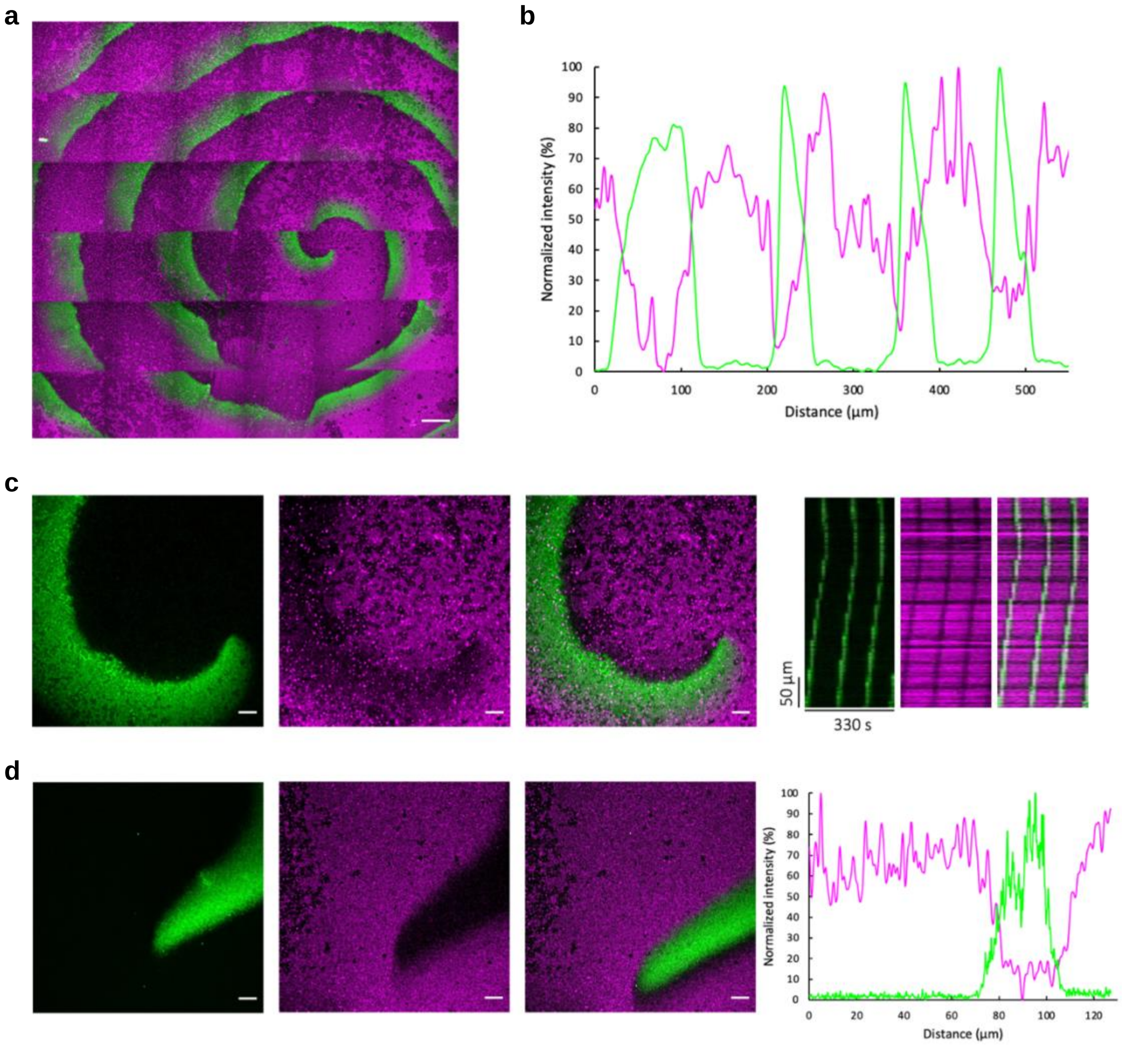

Figure S9. MinDE(C) proteins dynamically regulate ZipA-FtsZ patterns on supported membranes. **a** Mosaic of 6×6 tile scan microscope images showing large-scale organization of ZipA-FtsZ and MinDE dynamic patterns. MinDE oscillations are seen all over the chamber, co-existing and anti-correlating with ZipA-anchored FtsZ. Signals from eGFP-MinD and FtsZ-A647 are in green and magenta, respectively. A composite image of the overlaid channels is shown. Scale bar is 50 µm. **b** Intensity profiles of MinD and FtsZ signals. Color coding is the same as in **a**. **c** Fluorescence microscopy images of ZipA-anchored FtsZ and MinDE dynamic patterns. Signals from eGFP-MinD and FtsZ-A647 are in green and magenta, respectively. Scale bars are 10 µm. The corresponding kymographs are displayed on the right. **d** Fluorescence microscopy images of ZipA-FtsZ and MinDEC dynamic patterns. MinC stimulates redistribution of ZipA-anchored FtsZ filaments. Signals from eGFP-MinC and FtsZ-A647 are in green and magenta, respectively. Scale bars are 10 µm. The corresponding intensity profiles are shown on the right.

Table S1. List of primers used in this study

Name	Sequence (5' to 3')
173 ChD	CACACAGGAAACAGCTATGAC
174 ChD	GAGTCAGTGAGCGAGGAAG
365 ChD	CAGTCACGACGTTGTAAAACGAC
420 ChD	GGAGAGGCGGTTTGCGTAT
504 ChD	CTGCCCGCTTTCCAGTCGGGAAA
1139 ChD	CATGGTCATAGCTGTTTCCTGTGTGTAATACGACTCACTATAGG
1140 ChD	GGTTTCCCGACTGGAAAGCGGGCAGCAAAAAACCCCTCAAGACCCG
1145 ChD	CCGTCGTTTTACAACGTCGTGACTGGCGTTGGCCGATTCATTAATGC
1187 ChD	GGCGATTAAGTTGGGTAACG

Table S2. Transitions of the MS/MS measurements for the proteolytic peptides of the indicated proteins. Accelerator voltage was kept constant at 4 eV.

Protein	Compound name	Precursor ion (m/z)	Product ion (m/z)	Collision energy (eV)	Ion name
MinD	AYADTVER	462.725	690.342	15.3	y6
MinD	AYADTVER	462.725	619.305	15.3	y5
MinD	AYADTVER	462.725	304.162	15.3	y2
MinD	AYADTVER	462.725	235.108	15.3	b2
MinD	AYADTVER	462.725	306.145	15.3	b3
MinD	AYADTVER	462.725	621.288	15.3	b6
QconCAT	AYADTVER	468.208	699.315	15.3	y6
QconCAT	AYADTVER	468.208	627.281	15.3	y5
QconCAT	AYADTVER	468.208	309.147	15.3	y2
QconCAT	AYADTVER	468.208	237.102	15.3	b2
QconCAT	AYADTVER	468.208	309.136	15.3	b3
QconCAT	AYADTVER	468.208	627.270	15.3	b6
FtsA	YTELLNLVNEEILQLQEK	1095.089	1243.653	34.9	y10
FtsA	YTELLNLVNEEILQLQEK	1095.089	871.525	34.9	y7
FtsA	YTELLNLVNEEILQLQEK	1095.089	758.441	34.9	y6
FtsA	YTELLNLVNEEILQLQEK	1095.089	645.357	34.9	y5
FtsA	YTELLNLVNEEILQLQEK	1095.089	517.298	34.9	y4
FtsA	YTELLNLVNEEILQLQEK	1095.089	847.456	34.9	b7
QconCAT	YTELLNLVNEEILQLQEK	1106.555	1257.611	34.9	y10
QconCAT	YTELLNLVNEEILQLQEK	1106.555	881.495	34.9	y7
QconCAT	YTELLNLVNEEILQLQEK	1106.555	767.414	34.9	y6
QconCAT	YTELLNLVNEEILQLQEK	1106.555	653.333	34.9	y5
QconCAT	YTELLNLVNEEILQLQEK	1106.555	523.280	34.9	y4
QconCAT	YTELLNLVNEEILQLQEK	1106.555	855.432	34.9	b7
MinE	DILEVIC[+57]K	495.270	874.507	15.6	y7
MinE	DILEVIC[+57]K	495.270	761.423	15.6	y6
MinE	DILEVIC[+57]K	495.270	648.339	15.6	y5
MinE	DILEVIC[+57]K	495.270	471.245	15.6	b4
MinE	DILEVIC[+57]K	495.270	570.313	15.6	b5
MinE	DILEVIC[+57]K	495.270	683.397	15.6	b6
QconCAT	DILEVIC[+57]K	499.757	882.483	15.6	y7
QconCAT	DILEVIC[+57]K	499.757	768.402	15.6	y6
QconCAT	DILEVIC[+57]K	499.757	654.321	15.6	y5
QconCAT	DILEVIC[+57]K	499.757	475.233	15.6	b4
QconCAT	DILEVIC[+57]K	499.757	575.299	15.6	b5
QconCAT	DILEVIC[+57]K	499.757	689.380	15.6	b6
FtsA	GGVNDLESVVK	558.798	903.478	18.3	y8
FtsA	GGVNDLESVVK	558.798	789.435	18.3	y7
FtsA	GGVNDLESVVK	558.798	674.408	18.3	y6

Table S2. ***Continued***

Protein	Compound name	Precursor ion (m/z)	Product ion (m/z)	Collision energy (eV)	Ion name
FtsA	GGVNDLESVVK	558.798	561.324	18.3	y5
FtsA	GGVNDLESVVK	558.798	432.282	18.3	y4
FtsA	GGVNDLESVVK	558.798	214.119	18.3	b3
QconCAT	GGVNDLESVVK	565.279	913.449	18.3	y8
QconCAT	GGVNDLESVVK	565.279	797.412	18.3	y7
QconCAT	GGVNDLESVVK	565.279	681.388	18.3	y6
QconCAT	GGVNDLESVVK	565.279	567.306	18.3	y5
QconCAT	GGVNDLESVVK	565.279	437.267	18.3	y4
QconCAT	GGVNDLESVVK	565.279	217.110	18.3	b3
MinD	IIVVTSGK	408.763	703.435	13.7	y7
MinD	IIVVTSGK	408.763	590.351	13.7	y6
MinD	IIVVTSGK	408.763	491.282	13.7	y5
QconCAT	IIVVTSGK	413.250	711.411	13.7	y7
QconCAT	IIVVTSGK	413.250	597.330	13.7	y6
QconCAT	IIVVTSGK	413.250	497.265	13.7	y5
QconCAT	IIVVTSGK	413.250	429.300	13.7	b4
QconCAT	IIVVTSGK	413.250	531.345	13.7	b5
Ribosomal protein S4	LSDYGVQLR	525.783	850.442	17.3	y7
Ribosomal protein S4	LSDYGVQLR	525.783	735.415	17.3	y6
Ribosomal protein S4	LSDYGVQLR	525.783	572.351	17.3	y5
Ribosomal protein S4	LSDYGVQLR	525.783	635.304	17.3	b6
QconCAT	LSDYGVQLR	532.263	949.438	17.3	y8
QconCAT	LSDYGVQLR	532.263	861.409	17.3	y7
QconCAT	LSDYGVQLR	532.263	745.385	17.3	y6
QconCAT	LSDYGVQLR	532.263	581.325	17.3	y5
QconCAT	LSDYGVQLR	532.263	541.220	17.3	b5
QconCAT	LSDYGVQLR	532.263	641.286	17.3	b6
Ribosomal protein L6	APVVVPAGVDVK	575.845	883.525	18.9	y9
Ribosomal protein L6	APVVVPAGVDVK	575.845	784.456	18.9	y8
Ribosomal protein L6	APVVVPAGVDVK	575.845	685.388	18.9	y7
Ribosomal protein L6	APVVVPAGVDVK	575.845	268.166	18.9	b3
Ribosomal protein L6	APVVVPAGVDVK	575.845	367.234	18.9	b4
Ribosomal protein L6	APVVVPAGVDVK	575.845	466.302	18.9	b5
QconCAT	APVVVPAGVDVK	582.326	893.495	18.9	y9
QconCAT	APVVVPAGVDVK	582.326	793.430	18.9	y8
QconCAT	APVVVPAGVDVK	582.326	693.364	18.9	y7
QconCAT	APVVVPAGVDVK	582.326	271.157	18.9	b3
QconCAT	APVVVPAGVDVK	582.326	371.222	18.9	b4
QconCAT	APVVVPAGVDVK	582.326	471.288	18.9	b5
