# Supplementary material for: Min waves without MinC can pattern FtsA-anchored FtsZ filaments on model membranes
Source: Commun Biol. 2022 Jul 7;5:675. doi: 10.1038/s42003-022-03640-1 (PMC9262947; doi:10.1038/s42003-022-03640-1)
Supplement: Supplementary file 3 — Description of Additional Supplementary Files [file 42003_2022_3640_MOESM3_ESM.pdf]

Description of Additional Supplementary Files

File name: Supplementary Movie 1
Description: Video of FtsA-FtsZ and MinDEC dynamic patterns on a supported membrane. The experimental conditions are identical as in Fig. 2C. Page 7 of 17 Colors: eGFP-MinC (green), FtsZ-A647 (magenta). Scale bar is 10 µm.

File name: Supplementary Movie 2
Description: Video of FtsZ and MinDEC dynamic patterns without expressed FtsA on an SLB. In the absence of FtsA, FtsZ colocalizes with MinDEC waves. The experimental conditions are identical as in Fig. 2E. Colors: eGFP-MinC (green), FtsZA647 (magenta). Scale bar is 10 µm.

File name: Supplementary Movie 3
Description: Video of FtsA-FtsZ and MinDE dynamic patterns on an SLB. MinDE proteins, without MinC, regulate FtsA-FtsZ spatial organization. The experimental conditions are identical as in Fig. 3. Colors: eGFP-MinD (green), FtsZ-A647 (magenta). Scale bar is 10 µm.

File name: Supplementary Movie 4
Description: Videos of FtsA-FtsZ and MinDE dynamic patterns on an SLB for areas showing distinct rings and filaments of FtsZ. Waves effectively rearranging FtsA-anchored FtsZ filaments and low-amplitude propagating waves of FtsZ anticorrelating with MinDE patterns are played one after the other. The experimental conditions are identical as in Fig. 3. Colors: eGFP-MinD Page 8 of 17 (green), FtsZ-A647 (magenta). Scale bars are 10 µm

File name: Supplementary Movie 5
Description: Videos of MinDE(C) and FtsA-FtsZ exhibiting antiphase dynamic patterns in water-inoil droplets. One field of view is shown. The experimental conditions are identical as in Fig. 4. Colors: eGFP-MinC/D (green), FtsZ-A647 (magenta). Scale bars are 10 µm.

File name: Supplementary Movie 6
Description: Videos of two droplets exhibiting antiphase dynamic patterns of either MinDEC and FtsA-FtsZ (corresponding to the droplets in Fig. 4B), or MinDE and FtsZ (corresponding to the droplets in Fig. 4C), are played one after the other. The experimental conditions are identical as in Fig. 4. Colors: eGFP-MinC/D (green), FtsZ-A647 (magenta). Scale bars are 10 µm.

File name: Supplementary Movie 7
Description: Videos of droplets exhibiting antiphase dynamic patterns of MinDE(C) and FtsA-FtsZ (corresponding to the droplets in Fig. 4D, exact condition is as specified) are played side by side. The two droplets have a diameter >20 µm, allowing visualization of FtsZ cytoskeletal structures Page 9 of 17 extending to the lumen. The experimental conditions are identical as in Fig. 4. Colors: eGFPMinC/D (green), FtsZ-A647 (magenta). Scale bars are 10 µm.

File name: Supplementary Data 1
Description: Provides the mass spectrometrybased proteomics data, the Mathematica script for LC-MS/MS data analysis and the individual data points for the graphs in Fig. 1 B, C, Fig. S1C, Fig. S4D, and Fig. S5D.
