# Supplementary material for: Min waves without MinC can pattern FtsA-anchored FtsZ filaments on model membranes
Source: Commun Biol. 2022 Jul 7;5:675. doi: 10.1038/s42003-022-03640-1 (PMC9262947; doi:10.1038/s42003-022-03640-1)
Supplement: Supplementary file 12 — Reporting Summary [file 42003_2022_3640_MOESM12_ESM.pdf]

nature portfolio

Corresponding author(s): Christophe Danelon

Last updated by author(s): Jun 9, 2022

Reporting Summary

Nature Portfolio wishes to improve the reproducibility of the work that we publish. This form provides structure for consistency and transparency in reporting. For further information on Nature Portfolio policies, see our Editorial Policies and the Editorial Policy Checklist.

Statistics

For all statistical analyses, confirm that the following items are present in the figure legend, table legend, main text, or Methods section.

n/a	Confirmed	
☐	☒	The exact sample size (*n*) for each experimental group/condition, given as a discrete number and unit of measurement
☐	☒	A statement on whether measurements were taken from distinct samples or whether the same sample was measured repeatedly
☐	☒	The statistical test(s) used AND whether they are one- or two-sided<br>*Only common tests should be described solely by name; describe more complex techniques in the Methods section.*
☒	☐	A description of all covariates tested
☒	☐	A description of any assumptions or corrections, such as tests of normality and adjustment for multiple comparisons
☐	☒	A full description of the statistical parameters including central tendency (e.g. means) or other basic estimates (e.g. regression coefficient) AND variation (e.g. standard deviation) or associated estimates of uncertainty (e.g. confidence intervals)
☐	☒	For null hypothesis testing, the test statistic (e.g. *F*, *t*, *r*) with confidence intervals, effect sizes, degrees of freedom and *P* value noted<br>*Give P values as exact values whenever suitable.*
☒	☐	For Bayesian analysis, information on the choice of priors and Markov chain Monte Carlo settings
☒	☐	For hierarchical and complex designs, identification of the appropriate level for tests and full reporting of outcomes
☒	☐	Estimates of effect sizes (e.g. Cohen's *d*, Pearson's *r*), indicating how they were calculated

Our web collection on statistics for biologists contains articles on many of the points above.

Software and code

Policy information about availability of computer code

Data collection	LC-MS experiments: MassHunter Workstation Software LC/MS Data Acquisition for 6400 Series Triple Quadrupole Version B.07.01 Build 7.1.7112.0, Agilent Technologies, 2014.<br><br>Confocal microscopy: NIS Elements Version 4.51.01 Build 1146 LO, 64-bit, Nikon, 1991-2016.
Data analysis	MassHunter Workstation Software Quantitative Analysis Version B.06.00 Build 6.0.388.0, Agilent Technologies, 2008.<br><br>Microscopy image analysis was performed with FIJI Version 1.51 or MATLAB R2020b (MathWorks).<br><br>Data processing for LC-MS/MS analysis was performed with Mathematica (Wolfram Research, version 11.3).<br><br>Nonlinear regression of peptide concentration kinetics was performed with GraphPad Prism Version 8.2.1(279).<br><br>Statistical analysis of pattern properties was performed using a two-tailed Welch'st-test with GraphPad Prism Version 8.2.1(279). For null hypothesis testing, confidence interval was 95% and a degree of freedom of 14 was used.

For manuscripts utilizing custom algorithms or software that are central to the research but not yet described in published literature, software must be made available to editors and reviewers. We strongly encourage code deposition in a community repository (e.g. GitHub). See the Nature Portfolio guidelines for submitting code & software for further information.

Data

Policy information about availability of data

All manuscripts must include a data availability statement. This statement should provide the following information, where applicable:

- Accession codes, unique identifiers, or web links for publicly available datasets
- A description of any restrictions on data availability
- For clinical datasets or third party data, please ensure that the statement adheres to our policy

The authors declare that all data supporting the findings of this study are available within the article and its supplementary information files. The mass spectrometry-based proteomics data, the Mathematica script for LC-MS/MS data analysis and the individual data points for all graphs depicting a single point value are provided in the Source Data file. All materials and detailed protocols are available on request from the corresponding author.

Field-specific reporting

Please select the one below that is the best fit for your research. If you are not sure, read the appropriate sections before making your selection.

☒ Life sciences ☐ Behavioural & social sciences ☐ Ecological, evolutionary & environmental sciences

For a reference copy of the document with all sections, see nature.com/documents/nr-reporting-summary-flat.pdf

Life sciences study design

All studies must disclose on these points even when the disclosure is negative.

Sample size	No sample size calculation was performed. For all experiments, at least three samples were analyzed.
Data exclusions	No data was excluded from the analyses.
Replication	Reproducibility of the experimental findings was verified by preparing samples on different days and conducting the same experiments multiple times.
Randomization	Randomization was not relevant for this study.
Blinding	Blinding was not relevant for this study. No group allocation during data collection or analysis was performed.

Reporting for specific materials, systems and methods

We require information from authors about some types of materials, experimental systems and methods used in many studies. Here, indicate whether each material, system or method listed is relevant to your study. If you are not sure if a list item applies to your research, read the appropriate section before selecting a response.

Materials & experimental systems

n/a	Involved in the study	
☒	☐	Antibodies
☒	☐	Eukaryotic cell lines
☒	☐	Palaeontology and archaeology
☒	☐	Animals and other organisms
☒	☐	Human research participants
☒	☐	Clinical data
☒	☐	Dual use research of concern

Methods

n/a	Involved in the study	
☒	☐	ChIP-seq
☒	☐	Flow cytometry
☒	☐	MRI-based neuroimaging
